# Nine year in-hospital mortality trends in a high-flow level one trauma center in Italy

**DOI:** 10.1007/s13304-022-01303-8

**Published:** 2022-06-13

**Authors:** Elisa Reitano, Roberto Bini, Margherita Difino, Osvaldo Chiara, Stefania Cimbanassi

**Affiliations:** 1 Division of General Surgery, Department of Translational Medicine, Maggiore Della Carità Hospital, University of Eastern Piedmont, Corso Giuseppe Mazzini 18, Novara, Italy; 2 General Surgery and Trauma Team, ASST Niguarda, Piazza Ospedale Maggiore 3, 20162 Milan, Italy; 3 Emergency Department, Connolly Hospital Blanchardstown, Mill Rd, Abbotstown, Dublin, D15 X40D Ireland; 4 General Surgery and Trauma Team, University of Milan, ASST Niguarda, Piazza Ospedale Maggiore 3, 20162 Milan, Italy

**Keywords:** Trauma, Trauma center, Mortality distribution, Emergency medicine, Emergency surgery

## Abstract

Trauma is the leading cause of death in young people with a considerable socio-economic impact worldwide. A trimodal distribution of trauma mortality was described in the past, but recently different studies underlined a progressive change in trauma mortality distribution linked to improvement in trauma care. This study aimed to analyze the mortality trends in a Level-One Trauma Center in Italy. Data on 6065 patients consecutively admitted to the Trauma Center between 2011 and 2020 were selected and retrospectively analyzed. Causes of Death (CODs) and time of death were stratified in four main groups and the patient sample was further divided into five age groups. Multivariate regression models were then performed to identify independent predictors of mortality. The most common COD in all age groups was Central Nervous System injuries. Immediate deaths (in ED) affected mostly patients over 75 years of age (34.3%). Deaths caused by massive hemorrhage occurred soon upon arrival in the ED, whereas deaths due to other causes (e.g. sepsis, MOF) after the first week. Patients’ characteristics, the need for emergency procedures and high trauma severity scores were independent predictors of deaths. This study represented the first analysis on trauma mortality distribution in Italy over a nine-year period. The trimodal distribution described in the past seems to be no longer present in Italy, due to improvements in trauma systems and critical care. However, the high number of immediate and acute deaths underlies a persisting need for efforts in injury prevention and control .

## Introduction

Trauma is the first cause of death in younger people worldwide [1]. In 1983, Trunkey D [2] described a trimodal distribution of death in trauma patients. The first peak represented the immediate deaths, occurring within the first hour after trauma, accounting for 45% of all deaths, mostly due to non-survivable hemorrhagic or central nervous system injuries [2]. Early deaths, occurring within 1–4 h after trauma, represented the second peak and accounted for about 34% of deaths [3], mostly caused by uncontrolled hemorrhagic injuries or expanding intracerebral hematomas. If these injuries are promptly recognized and treated, early deaths can be prevented: therefore these deaths have also been defined as preventable trauma deaths [4]. The third peak occurred more than a week after trauma and accounted for about 20% of all deaths, defined as late deaths [3]. Most of these deaths were attributed to sepsis and multiple organ failure (MOF) [4].

The concept of a trimodal distribution of death in trauma played an important role in the development of trauma systems. The development of damage control techniques, specific guidelines in massive transfusion protocols, and the implementation of modern technologies in intensive care has decreased the mortality of trauma patients [5, 6], leading to a bimodal distribution of trauma mortality [7, 8]. The increase in the age of the trauma population, as well as the presence of comorbidities and the predominance of some mechanisms of injury (MOIs), contributed to the change in the epidemiology of trauma related deaths over the past decade [9–11].

Different authors investigated the role of MOI, cause of death (COD), and time of death [7, 8] on the distribution of mortality as the relationship between these factors could be relevant in the assessment of the effectiveness of a trauma system. The aim of this study was to investigate the mortality distribution in a high-flow Level One Trauma Center in Italy over a nine-year period. To our knowledge, this is the first study that describes the distribution of trauma deaths and the effect of the introduction of a mature trauma system in Italy.

## Methods

All major trauma managed in Niguarda Trauma Center were prospectively collected in Niguarda trauma registry from 2011 to 2020. The institution of the trauma registry for all major trauma admitted to the hospital was approved by Niguarda Ethical Committee Milano Area 3 (record number 534–102,018). Accordingly, no personal data of patients are disclosed in the present study. The registry is held by a Trauma Team consultant who is meant to keep it constantly updated, and it is annually revised by the head of the department. Demographic data, time of trauma, vital parameters (heart rate, respiratory rate, systolic blood pressure, Glasgow Coma Scale (GCS), MOI, emergency procedures performed in the emergency department (ED), need of emergency surgery, need of orotracheal intubation (IOT), and injuries sustained by the patients were extracted from the registry. The abbreviated injury scale (AIS, 1998 version) of each anatomical region, Injury Severity Score (ISS), Revised Trauma Score (RTS), probability of survival (PS) obtained by the Revised Trauma and Injury Severity Score (TRISS) system, and observed survival were retrospectively analyzed to estimate the severity of the trauma. The American Society of Anesthesiologists (ASA) physical status classification was chosen to summarize comorbidities. Patients were divided into five age groups: pediatric age (0–13 years), young adults (14–39 years old), adults (40–64 years old), elder adults (65–75 years old) and elderly (> 75 years old). Patients with ISS ≥ 16 were considered severely injured.

CODs were classified as follows:

- Central Nervous System (CNS): predominantly lethal injury of brain, brain stem, and high cervical spine.
- Massive hemorrhage (HEM): hemorrhage, predominantly from uncontrolled bleeding “clinically visualised or otherwise documented (disruption of large vessels or parenchymatous organ leading to complete loss of blood volume, or hypovolemic cardiac arrest)” [12].
- CNS + HEM: combination of the previous two.
- Other: MOF, ARDS, other injuries or secondary complications (pulmonary embolism, sepsis, myocardial infarction).

Deceased patients were also stratified over four groups based on the time of death:

- Immediate: patients deceased early after admission in the ED to whom no patient ID number was assigned;
- Acute: death within 24 h from access in the ED;
- Early: death after 24 h but within 7 days;
- Late: demise of the patient after 7 days from arrival in the ED

The data were recorded in a computerized spreadsheet (Microsoft Excel 2016, Microsoft Corporation, Redmond, WA) and analyzed with statistical software (IBM Corp. Released 2012, IBM SPSS Statistics for Windows, version21.0, Armonk, NY, IBM Corp.). Graphics were also obtained with R coding. The sample distribution was evaluated with Kolmogorov–Smirnov and Shapiro–Wilk tests, resulting in a non-Gaussian distribution for any of the examined variables. Continuous data were compared by independent sample Kruskar–Wallis test, and categorical data using Pearson’s chi-square test. P values below 0.05 were considered statistically significant, and included in the multivariate logistic regression model. Survival curves were obtained with Kaplan–Meier analysis, and log-rank test was assessed to evaluate differences in cumulative survival among age groups. Bivariate logistic regression was used to provide odds ratio for individual variables, identifying possible predictors of mortality. After computing the variance inflation factor (VIF), showing no collinearity, three different multivariate regression models were built: one for general variables (age, in-hospital vital parameters), another one for the pre-hospital and in-hospital maneuvers and surgeries performed and the last one for the injury’s severity indicators (AIS’98, ISS, TRISS) to detect independent risk factors for death and to estimate the adjusted odds ratio (OR) and 95% confidence interval (CI).

## Results

During the study period, of the 6065 patients who fulfilled the inclusion criteria, 316 (5.21%) died. Table 1 resumes the univariate and multivariate analysis among the general population. Significant variables which correlated with mortality using bivariate logistic regression analysis were Age, ASA score, SBP, DBP, GCS, RTS, BE levels, extra-peritoneal packing (EPP), damage control laparotomy (DCL) and thoracotomy (DCT), thoracic drain placement, need of emergency surgery and IOT, ISS and TRISS.

**Table 1:** General characteristics among the groups

	Univariate analysis			Logistic regression model		
	Survived (*n* = 5749)	Dead (*n* = 316)	*P* value	*P* value	Adjusted OR	95% CI (Lower–upper)
Male [*n* (%)]	4244 (73.8)	229 (72.5)	0.595			
Age [Median (IQR)]	37 (24–52)	59 (40–78)	≤ 0.001*	≤ 0.001*	1.050	1.041–1.058
ASA Score ≥ 3 [Median (IQR)]	200 (3.9)	45 (15.5)	≤ 0.001*	≤ 0.001*	0.256	0.168–0.390
HR ED [Median (IQR)]	86 (75–100)	87 (64.25–112.25)	0.832			
SBP ED [Median (IQR)]	130 (119–145)	94 (60–130)	≤ 0.001*	0.002*	0.986	0.977–0.995
DBP ED [Median (IQR)]	78 (70–85)	60 (35–80)	≤ 0.001*	0.026*	0.983	0.968–0.998
GCS ED [Median (IQR)]	15 (15–15)	3 (3–3)	≤ 0.001*	0.006*	0.883	0.807–0.966
BE [Median (IQR)]	−1.50 (−3.7 to 0.50)	−7.2 (−13.20 to 3.20)	≤ 0.001*	≤ 0.001*	0.827	0.802–0.852
INR ratio [Median (IQR)]	1.06 (1.00–1.13)	1.36 (1.11–1.88)	≤ 0.001*	0.521	1.006	0.987–1.026
Lactate [Median (IQR)]	1.98 (1.40–2.80)	5.30 (2.88–9.85)	≤ 0.001*	0.843	1.00	0.996–1.003
EPP [*n *(%)]	50 (0.9)	42 (13.3)	≤ 0.001*	0.002*	0.358	0.189–0.678
DCL [*n *(%)]	168 (2.9)	41 (13)	≤ 0.001*	0.881	0.955	0.525–1.740
DCT [*n *(%)]	3 (0.1)	8 (2.5)	≤ 0.001*	0.005*	0.083	0.014–0.476
Right thoracic drain [*n* (%)]	185 (3.2)	44 (13.9)	≤ 0.001*	0.001*	0.482	0.320–0.728
Left thoracic drain [*n *(%)]	182 (3.2)	58 (18.4)	≤ 0.001*	≤ 0.001*	0.245	0.169–0.354
Emergency Surgery [*n *(%)]	1524 (26.5)	157 (49.7)	≤ 0.001*	≤ 0.001*	0.451	0.356–0.572
Interventional radiology [*n *(%)]	186 (3.2)	29 (9.2)	≤ 0.001*	0.365	0.773	0.422–1.350
Massive transfusion [*n *(%)]	401 (7)	141 (44.6)	≤ 0.001*	0.105	2.718	0.813–9.816
IOT [*n *(%)]	836 (14.5)	245 (77.5)	≤ 0.001*	≤ 0.001*	0.127	0.094–0.172
Head AIS’98 ≥ 3 [*n *(%)]	1021 (36.6)	241 (92)	≤ 0.001*	0.055	0.290	0.082–1.027
Face AIS’98 ≥ 3 [*n *(%)]	66 (6.5)	16 (15.8)	0.001*	0.233	0.504	0.163–1.555
Chest AIS’98 ≥ 3 [*n *(%)]	1394 (76.2)	198 (93)	≤ 0.001*	0.426	0.523	0.106–2.578
Abdomen AIS’98 ≥ 3 [*n *(%)]	423 (36.6)	67 (56.3)	≤ 0.001*	0.793	1.117	0.490–2.544
Extremity AIS’98 ≥ 3 [*n *(%)]	1071 (40.1)	106 (67.1)	≤ 0.001*	0.856	0.926	0.403–2.126
ISS [Median (IQR)]	8 (1–17)	41 (26–59)	≤ 0.001*	≤ 0.001*	1.083	1.072–1.094
ISS ≥ 16 [*n* (%)]	1731 (30.1)	307 (97.2)	≤ 0.001*	≤ 0.001*	0.034	0.017–0-067
RTS [Median (IQR)]	12 (12–12)	4 (2–7)	≤ 0.001*	0.027*	0.874	0.776–0.985
Probability of death (TRISS'98) [Median (IQR)]	0.70 (0.40–3.0)	93.95 (76.10–98.70)	≤ 0.001*	≤ 0.001*	1.072	1.047–1.097

*ASA* American society of anesthesiologists, *HR* heart rate, *SBP* systolic blood pressure, *DBP* diastolic blood pressure, *GCS* glasgow coma scale, *BE* basic excess, *INR* international normalized ratio, *EPP* extra-peritoneal pelvic packing, *DCL* damage control laparotomy, *DCT* damage control thoracotomy, *IOT* orotracheal intubation, *AIS* abbreviated injury scale, *ISS* injury severity score, *RTS* revised trauma score, *TRISS* trauma and injury severity score

*Statistical significance

Table 2 resumes the COD according to the mechanism of trauma and intent among the 316 deceased patients. Deaths in motorcycle and motor vehicle collisions were mostly caused by CNS (45.5% and 44.7% respectively) and HEM (34.1% and 23.7%). Deaths in accidental falls were related to CNS in 73.3% of cases, and CNS + HEM in 15.6% of cases. The highest ratio of death due to ‘other’ causes was found in bicycle related injuries (18%, two third of which were elderly). Most deceased cyclists (*n *= 14.42%) were over 75 years of age. The most common COD in all age groups was CNS. ‘Other’ causes of death were found in almost 14% of elderly (> 75) and in lower ratios in the other age groups. Among those who died of ‘other’ causes, only *one* patient had less than 41 years of age.

**Table 2:** Causes of dead per moi and intent

MOI [*n *(%)]	CNS	HEM	CNS + HEM	Other	Tot
Fall	51 (47.2)	23 (21.3)	27 (25.0)	7 (6.5)	108
Pedestrian	31 (53.4)	13 (22.4)	8 (13.7)	6 (10.3)	58
MCC	20 (45.5)	15 (34.1)	9 (20.5)	–	44
MVC	17 (44.7)	9 (23.7)	7 (18.4)	5 (13.2)	38
Bicycle	22 (66.7)	1 (3)	4 (12.1)	6 (18.2)	33
GSW	12 (92.3)	–	–	1 (7.7)	13
SW	–	7 (87.5)	–	1 (12.5)	8
Crush injury	–	1 (50)	1 (50)	–	2
Other	11 (100)	–	–	–	11
NN	1 (100)	–	–	–	1
ICD9 Categories of trauma [*n*(%)]					
Road traffic	90 (51.7)	39 (22.4)	28 (16.1)	17 (9.8)	174
Work related	9 (69.2)	1 (7.7)	3 (23.1)	–	13
Accidental fall	33 (73.3)	2 (4.4)	7 (15.6)	3 (6.7)	45
Self-inflicted	16 (45.7)	13 (37.1)	4 (11.4)	2 (5.7)	35
Assault	6 (54.5)	4 (36.4)	–	1 (9.1)	11
NN	11 (28.9)	10 (26.3)	14 (36.8)	3 (7.9)	38

*MOI* mechanism of injury, *CNS* central nervous system, *HEM* massive hemorrhage, *MCC* motorcycle collision, *MVC* motor vehicle collision, *SW* stab wound, *GSW* gunshot wound

However, head AIS was not found to be predictor of death at the multivariate analysis. Deaths due to a combination of CNS HEM occurred in patients with higher ISS and at a younger age.

Table 3 resumes the time of death in the different age groups. Immediate deaths (in ED) affected mostly patients over 75 years of age (34.3%). Table 4 describes the time of death in the different COD groups. Deaths caused by massive hemorrhage occurred soon upon arrival in the ED, whereas deaths due to ‘Other’ causes occurred later in time, after the first week (Fig. 2).

**Table 3:** Time of death per age group

Time of death						
Age groups [*n* (%)]	Immediate (in ED)	Acute (< 24 h)	Early (2–7 days)	Late (> 7 days)	Unknown	Total
0–13	5 (5.2)	3 (3.3)	2 (2.1)	–	–	10
14–39	20 (21.0)	26 (28.8)	15 (16.3)	3 (7.9)	–	64
40–64	31 (32.6)	25 (27.8)	28 (30.5)	17 (44.7)	–	101
65–75	6 (6.4)	15 (16.7)	20 (21.7)	5 (13.2)	1 (100)	47
> 75	33 (34.8)	21 (23.4)	27 (29.4)	13 (34.2)	–	94
Total	95	90	92	38	1	

*ED* emergency department

**Table 4:** Time of death per cause of death

Time of death					
COD	Immediate (in ED)	Acute (< 24h)	Early (2–7 days)	Late (>7 days)	Total
CNS *n *(%)	45 (48.3)	32 (35.5)	74 (80.4)	13 (34.2)	164
CNS + HEM *n *(%)	15 (16.1)	29 (32.2)	8 (8.7)	2 (5.3)	56
HEM *n *(%)	32 (34.4)	29 (32.2)	5 (5.4)	3 (7.9)	69
Other	1 (1.0)	0	5 (5.4)	20 (52.6)	26

*COD* cause of death, *CNS* central nervous system, *HEM* massive hemorrhage, *ED* emergency department

**Fig. 1:**
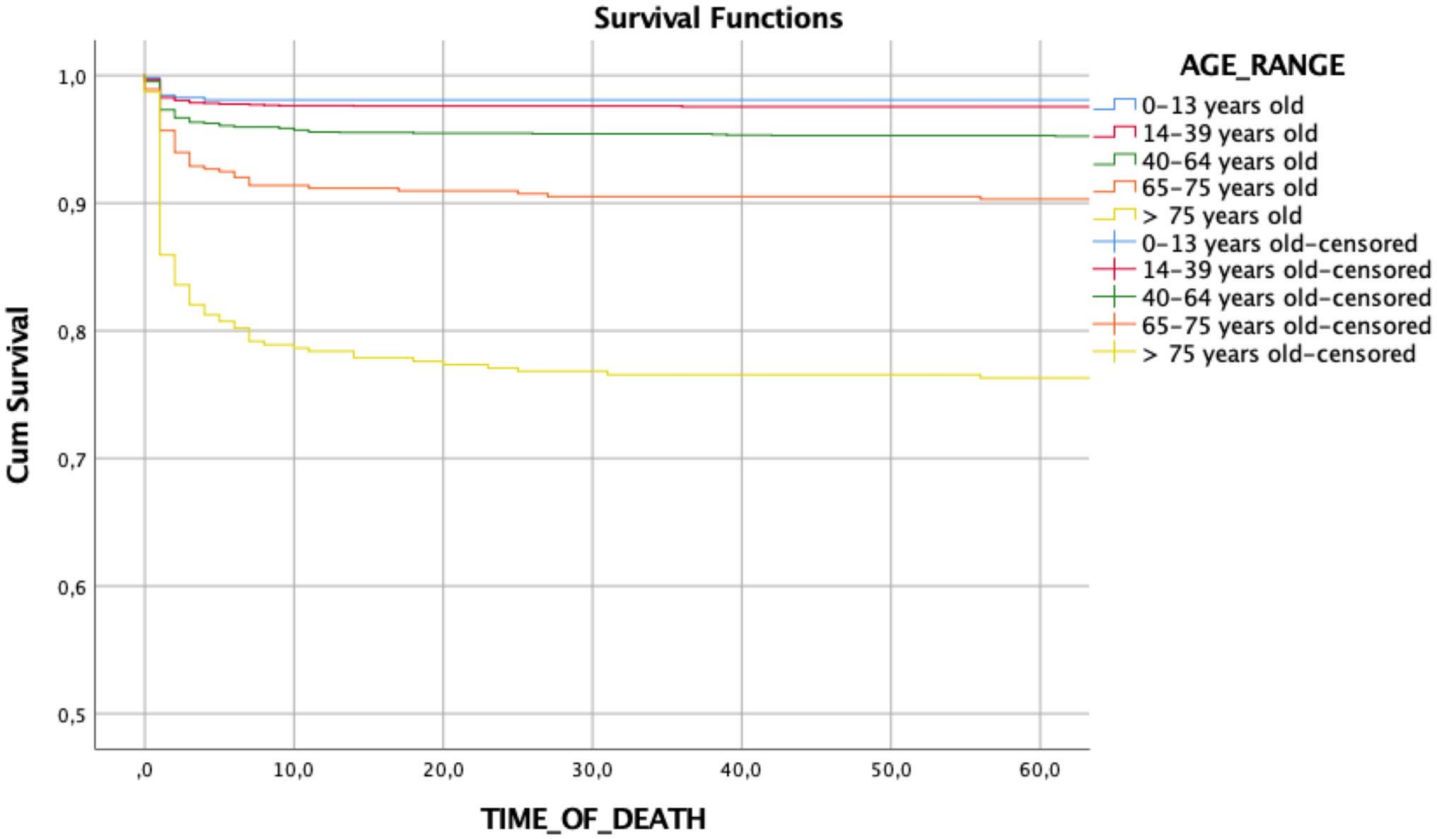
Kaplan–Mayer survival curves among different age groups

The survival rate computed with Kaplan–Meier method was 98.1% in patients younger than 13 years of age. In patients between 14 and 39 years it was 97.6%, 95.1% in patients between 40 and 64 years, 89.9% in patients between 65 and 75 years and 75% in patients over 75 years of age (log-rank test, *p* < 0.001). Survival curves among the different age groups are reported in Fig. 1.

## Discussion

Our study represented the first analysis on trauma mortality distribution in Italy over a nine-year period. The classic trimodal distribution of trauma mortality, previously described in literature, was no longer present. Improvements in trauma systems and critical care changed the distribution of mortality, with most deaths occurring within the first hour and an important decrease in the number of late deaths: patients who survived the first hours were more likely to survive. The persisting elevated number of immediate and acute deaths underlined the need for further efforts in injury prevention and control. In a mature trauma system immediate and acute deaths affected principally patients unlikely to survive due to the severity of the injuries who arrived alive in the ED because of the efficiency of the pre-hospital care.

Multivariate logistic regression (Table 1) showed that patient characteristics (such as age, ASA score and vital parameters), the need for Damage Control procedures (EPP, DCT, thoracic drain placement, emergency surgery etc.) and trauma severity scores (ISS, death probability TRISS, RTS), were independent predictors of mortality. Therefore, mortality seemed to be related to different factors, with a different impact according to the timing of treatment. Kaplan Mayer analysis (Fig. 1) showed that mortality was higher in patients older than 75 years old with a greater incidence of immediate and acute deaths, thus confirming the impact of age on mortality. These results were in line with recent international literature [7, 13]. However, Table 1 shows how variables often associated with an increased mortality (such as emergency surgery needing or ISS > 16) were not independent predictors of mortality. In our study, “emergency surgery” included any emergency procedure performed in the operating room immediately after the patient arrival. Therefore, also non-life-saving procedure (as orthopedics or plastic procedures) are included in this definition. This can explain how this variable was not a predictor of mortality. Concerning the ISS > 16, this study was conducted in a high-flow trauma center with a specialized trauma team consisting of surgeons, anesthesiologist, and radiologist immediately available at the trauma arrivals. It is therefore possible that these data represent the protective effect of an experienced trauma team in reducing mortality in seriously injuries patients.

**Fig. 2:**
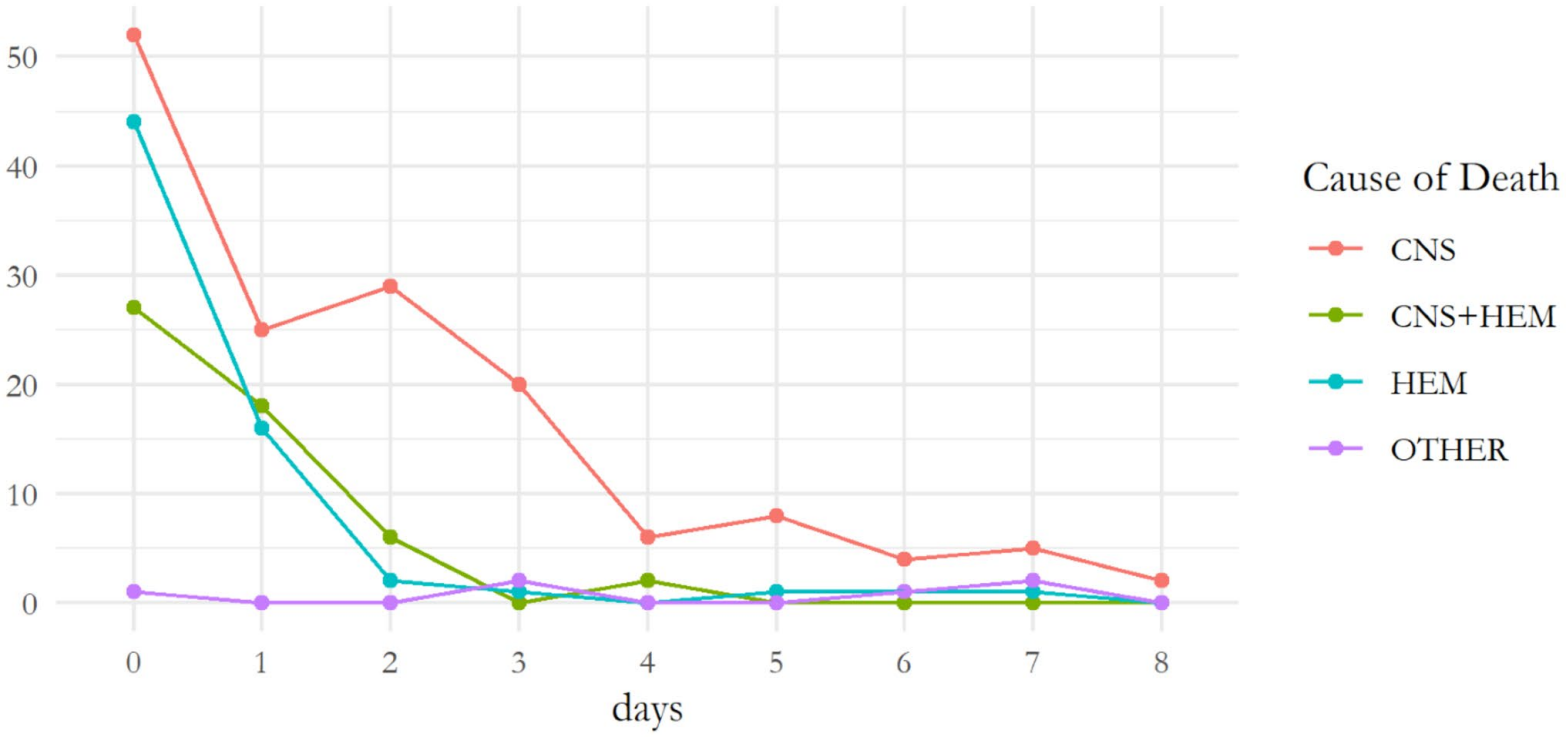
Cause of death during the first 8 days

Figure 1, 2, shows a decreasing in mortality over the time with a clear decrease of trauma mortality in late deaths. Decrease in late deaths could be ascribed to two main reasons: (a) the efficacy of damage control surgery and resuscitation in the acute phase [14], with early use of blood products, application of massive transfusion protocols [5] and on the minimization of crystalloid resuscitation [15]; (b) improvement in intensive care treatment and artificial support which allowed for an increased survival of sick patients. Late deaths, occurring days to weeks after trauma, were mainly related to hypovolemic shock and massive crystalloid resuscitation, resulting in ischemia–reperfusion mechanism with cellular damage and multiple organ disfunction (such as cardiac failure, acute renal failure, acute respiratory distress syndrome, infection and sepsis) [16, 17]

Table 2 shows the mortality distribution according to the MOI and intent. Falls represented the first cause of death, followed by pedestrian struck and motorcycle accidents, with CNS injuries as the first cause of mortality in these groups. Lansink et al. [18] analyzed the trauma distribution in a Level One Trauma Center in Germany, showing a great incidence of blunt trauma, with CNS injuries as a main COD, but HEM playing a major during the first hour after trauma. These results diverged from North American reports where HEM is the first COD in all stages, as the main MOI are gunshot wounds (GSW) and stab wounds (SW) [19].

In contrast with North American Literature [7, 20], GSW and SW represented a minor cause of death in our sample: penetrating injuries represented only approximately 8% of all injuries, and most GSWs were self-inflicted in male patients > 40 years of age. These results were in line with the overall trauma trends, as the incidence of this type of MOIs is higher in North America than in Europe [11, 20]. Our study was conducted in a high-flow Level One trauma center and showed, for the first time in Italy, the potential role of a specialized and trained Trauma Team in reducing mortality. The reduction in mortality is mainly linked to HEM through the damage control maneuvers performed in emergency room. Indeed, in the first hours upon arrivals, CSM represented the main cause of death.

Some limitations must be considered when interpreting the results present in this study. First of all, only intra-hospital data were analyzed, thus not providing information on pre-hospital mortality. The development of a mature trauma system, especially in the urban area, led to a “scoop and run” approach in the pre-hospital setting. Therefore, it is possible that many of the patients who would have previously been declared dead on the scene were transported to the hospital, increasing the number of immediate deaths in the ED due to non-survivable injuries, such as severe CNS injuries. Further studies are needed to confirm this finding.

In addition, the study was a retrospective analysis of a nine-year cohort of trauma patients transported and treated in a level one trauma center: a high-flow trauma center with a surgical leadership in trauma management and a staff specifically trained on trauma management and damage-control procedures [11, 21]. Although it represented the first Italian report on the topic, it cannot reflect the overall mortality distribution of trauma patients in all the country, as there are differences in trauma management according to the center. Further multi-center studies should be carried out to confirm these results.

## Conclusions

This study confirms that the trimodal distribution of trauma mortality is no longer present in Italy. Advances in trauma management, development of evidence-based protocols for acute care of injuries, multidisciplinary care of the injured and primary social measure for trauma prevention probably contributed to the changes in the observed distribution of trauma mortality. Moreover, this study showed a significant difference in mortality distribution in Italy with a higher incidence of blunt road traffic trauma and CNS injuries as main COD comparing with North American literature. Further multi-center studies also including the pre-hospital data should be carried out to confirm and expand our results.
